# Supplementary material for: The Oral Transglutaminase 2 Inhibitor ZED1227 Accumulates in the Villous Enterocytes in Celiac Disease Patients during Gluten Challenge and Drug Treatment
Source: Int J Mol Sci. 2023 Jun 28;24(13):10815. doi: 10.3390/ijms241310815 (PMC10341493; doi:10.3390/ijms241310815)
Supplement: Supplementary file 1 [file ijms-24-10815-s001.zip › ijms-2427768-supplementary.pdf]

Supplementary Materials

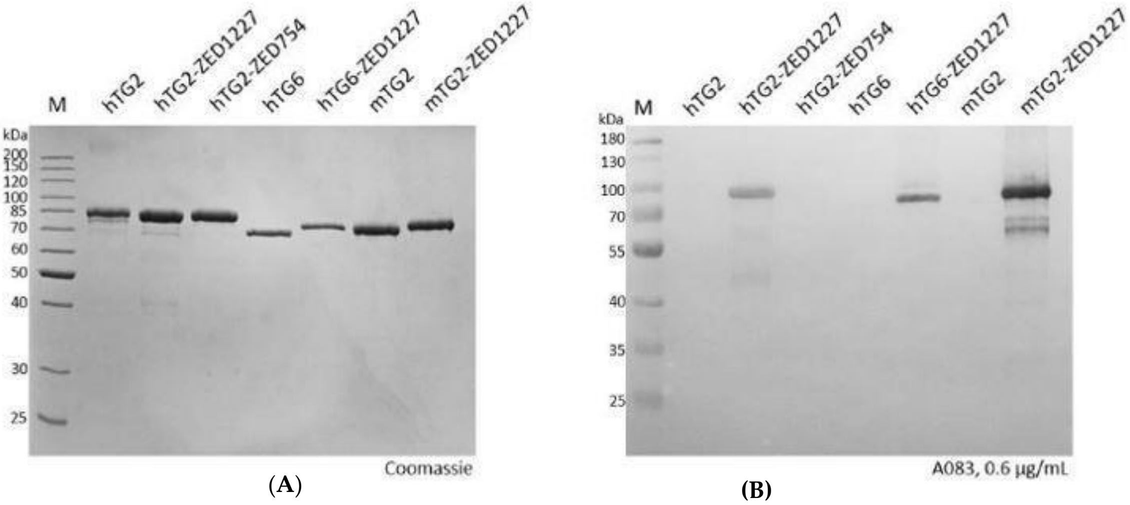

(A) (B)

Figure S1. Coomassie stained SDS-PAGE gel (panel A) of human TG2 (hTG2), hTG2-ZED1227 conjugate, hTG2-ZED754 (peptidic lead structure; Buechold et al., 2022), human TG6, hTG6-ZED1227 conjugate, mouse TG2 (mTG2), and mTG2-ZED1227 conjugate. Western blot (panel B) using the monoclonal antibody A083 (0.6 ug/ml). Only conjugates containing ZED1227 were detected by the antibody.

(A) (B) (C)

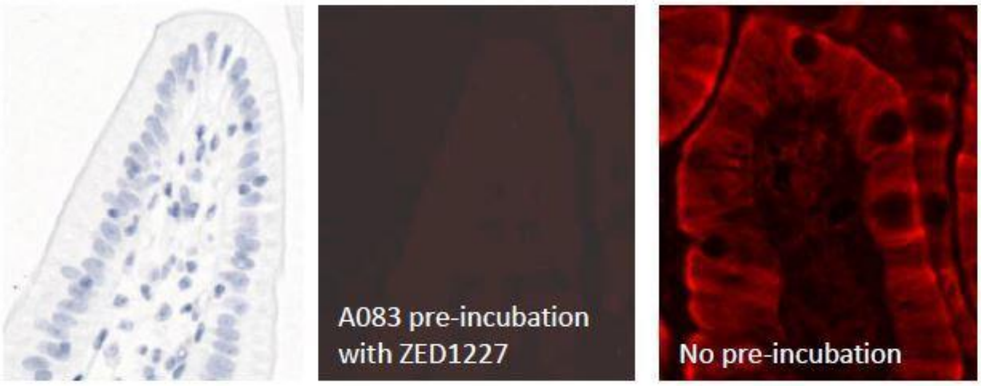

Anti-ZED1227 antibody pre-incubation with ZED1227 before applying on the tissue section for immunofluorescence staining.

Staining is abolished indicating specificity of the labeling to ZED1227.

Figure S2. immunofluorescence specificity control with ZED1227 antibody (A083) pre-incubated with ZED1227 before applying on the tissue section for immunostaining. Staining is abolished indicating specificity of the labeling to ZED1227 (compare panels B and C). Panel A shows the counterstaining of panel B. Magnification, x400.

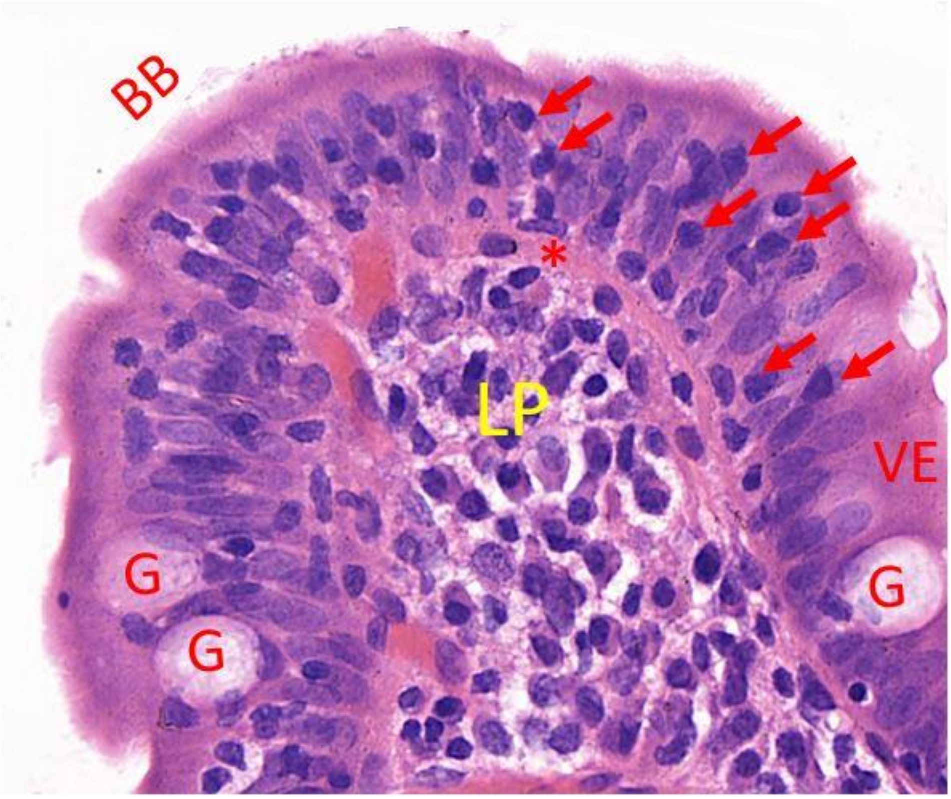

Figure S3. Histological structure of duodenal villus tip from a celiac patient. H&E staining, x 400. Annotations: VE=villus epithelium; BB=Brush border; Arrows show intraepithelial lymphocytes (cells with small dark round nuclei). Asterisk marks the basement membrane; G=Goblet cells L P=Lamina propria.
